# Measuring Nurses’ Knowledge and Awareness of Climate Change and Climate-Associated Diseases: Protocol for a Systematic Review of Existing Instruments

**DOI:** 10.3390/ijerph20206963

**Published:** 2023-10-23

**Authors:** Omar Portela Dos Santos, Pauline Melly, Stéphane Joost, Henk Verloo

**Affiliations:** 1 Department of Nursing Sciences, School of Health Sciences, HES-SO Valais/Wallis, University of Applied Sciences and Arts Western Switzerland, 1950 Sion, Switzerland; pauline.melly@hevs.ch (P.M.); henk.verloo@hevs.ch (H.V.); 2 Institute of Health Sciences, Universidade Católica Portuguesa, 4169-005 Porto, Portugal; 3 Geospatial Molecular Epidemiology Group (GEOME), Laboratory for Biological Geochemistry (LGB), Ecole Polytechnique Fédérale de Lausanne (EPFL), 1015 Lausanne, Switzerland; stephane.joost@epfl.ch; 4 Unit of Population Epidemiology, Division and Department of Primary Care Medicine, Geneva University Hospitals, 1205 Geneva, Switzerland; 5 La Source School of Nursing, University of Applied Sciences and Arts Western Switzerland (HES-SO), 1004 Lausanne, Switzerland; 6 Department of Psychiatry, Lausanne University Hospital, Service of Old Age Psychiatry, 1008 Lausanne, Switzerland

**Keywords:** climate change, global warming, environment and public health, health literacy, information literacy, eco-literacy, nurses, nursing students

## Abstract

Background: Climate change is a health emergency. Each year, it is estimated to cost more than 230 million years of life expectancy, with 4–9 million premature deaths associated with air pollution, and 9 million excess deaths due to non-optimal temperatures, representing 7% more temperature-related deaths since 2015 and 66% more since 2000. Objective: Identify and evaluate the reliability, fidelity, and validity of instruments measuring nurses’ knowledge and awareness of climate change and climate-associated diseases. Methods: A systematic literature review will retrieve and assess studies examining instruments measuring nurses’ knowledge and awareness of climate change and climate-associated diseases. Using predefined search terms for nurses, climate change, literacy and scales or tools, we will search for published articles recorded in the following electronic databases, with no language or date restrictions, from their inception until 31 October 2023: Medline Ovid SP (from 1946), PubMed (NOT Medline[sb], from 1996), Embase.com (from 1947), CINAHL Ebesco (from 1937), the Cochrane Library Wiley (from 1992), Web of Science Core Collection (from 1900), the Trip Database (from 1997), JBI OVID SP (from 1998), and the GreenFILE EBSCO. We will also hand-search relevant articles’ bibliographies and search for unpublished studies using Google Scholar, ProQuest Dissertations and Theses Global, and DART-EUrope.eu. This will be completed by exploring the gray literature in OpenGrey and the Grey Literature Report, from inception until 31 October 2023, in collaboration with a librarian. Twelve bibliographic databases will be searched for publications up to 31 October 2023. The papers selected will be assessed for their quality. Results: The electronic database searches were completed in May 2023. Retrieved articles are being screened, and the study will be completed by October 2023. After removing duplicates, our search strategy has retrieved 3449 references. Conclusions: This systematic review will provide specific knowledge about instruments to measure nurses’ knowledge, awareness, motivation, attitudes, behaviors, beliefs, skills, and competencies regarding climate change and climate-associated diseases.

## 1. Introduction

The World Health Organization (WHO) defines a disaster as an event that causes damage, ecological disruption, threats to human life, or the deterioration of human health and health services [1]. A disaster’s impact is determined by (i) the type of hazard itself, (ii) the vulnerability of the affected population, and (iii) the capacity to cope with its negative consequences [1]. Predictions about climate change suggest alarming consequences [2,3]. Since 2019, climate change has become a health threat of the same magnitude as non-communicable and cardiovascular diseases [4]. Raising awareness and changing behaviors have become urgent [5], as have health promotion and prevention measures [2]. Nurses, as health educators and social actors, must be proactive, playing a central role in creating change and improving planetary health [6]. Full planetary health is the highest level of health, well-being, and equity, achieved through awareness and the thoughtful management of the political, economic, and social systems [7]. To accomplish this, nurses’ eco-literacy will require significant improvements, i.e., their awareness, skills, and knowledge about new, climate-induced health parameters.

According to the WHO, nine out of ten people in the world breathe poor-quality air. Furthermore, non-optimal temperatures are responsible for nine million excess deaths annually—an increase of 7% since 2015 and 66% since 2000 [8]. These numbers are continuing to evolve [9]. Indeed, greenhouse gas emissions, such as carbon dioxide (CO_2_), nitrous oxide (N_2_O), and methane (CH_4_), have been rising since the 1850s. More than 230 million years of human life expectancy is lost each year due to polluting emissions [3]. Of all the unwanted waste of human origin released into the air, land, water, and oceans [9], particulate matter (PM), nitrogen dioxide (NO_2_), ground-level ozone (O_3_), and sulfur dioxide (SO_2_) are the pollutants with the greatest impacts on human health [10]. These gases affect the frequencies of extreme weather events and droughts that can lead to a lack of groundwater, limited food availability, rising sea levels, and melting polar ice sheets [11]. The European healthcare sector emits 4.4% of all global emissions, making a major contribution to the climate crisis [10,12]. Average European air temperatures are now 2.2 °C higher than pre-industrial levels, which is 1 °C above the average global temperature increase. The hottest European summer was in 2022 [13]. Since 2010, exposure to heat waves has increased by 57% and by more than 250% in some regions [13,14]. Exposure to suboptimal ambient temperatures is among the most significant environmental health hazards [8].

Climate change causes many direct and indirect negative health effects, including physical and mental disorders. Its direct health effects are often related to age, pre-existing illnesses, and co-morbidities, whereas its indirect effects, mediated through changes in the biosphere, include mood disturbances, irritability, anxiety, physical weakness, pains, insomnia, heart failure, malnutrition, dehydration, asthma, cancers, myocardial infarction, or even stroke [2,13,15]. Furthermore, climate change exacerbates existing inequalities in disease burden and premature mortality [3,16]. Environmental risk factors are responsible for 80% of common diseases and 25–33% of the total disease burden [5]. Older adults (people aged 65 or older), infants (up to 1 year old), people with chronic diseases (e.g., cardiovascular disease, respiratory disease, kidney disease, diabetes), pregnant women, those living in urban environments at higher altitudes, and people with low socioeconomic status or living in social isolation [8] are considered the most vulnerable population groups [6,17,18].

In Switzerland, the consequences of climate change are also having an impact on the healthcare system, with an increase in emergency room visits and healthcare costs. Extreme temperatures may be a risk factor for heat stroke, heat edema, heat rash, heat stress, acute cardiovascular disease, electrolyte dysfunction, the exacerbation of chronic respiratory and cardiovascular diseases, renal disease, pneumonia, certain infectious diseases, and diseases of the genitourinary system [6,18]. Ambient air pollution is also a considerable risk to the respiratory system by directly promoting or aggravating respiratory diseases or increasing exposure to risk factors. The multi-country time-series analysis conducted by Liu et al. [19], in 652 urban areas in 24 countries, indicated that an increase of 10 μg per cubic meter in the PM_10_ concentration was associated with an increase of 0.36% in daily cardiovascular mortality and an increase of 0.47% in daily respiratory mortality. Exposure to ambient air pollution and extreme temperatures is also associated with increased hospital admissions. Tobaldini et al. [20] found a higher risk of out-of-hospital cardiac arrest (OHCA) events with higher levels of particulate matter. Stronger associations were found on warmer days, suggesting that ambient temperature positively contributes to the OHCA risk associated with high levels of PM. Finally, the scoping review of 22 studies conducted by Cicci et al. [21] indicated positive relationships between high temperatures and total cardiovascular diseases and ischemic heart diseases, acute myocardial infarction, congestive heart failure risk (when pre-existing heart failure is diagnosed), as well as stroke ED visits and hospitalizations.

Nowadays, climate change must be a key concern for the nursing discipline. Thanks to daily practice, research, and training, nurses can provide effective responses to the impacts of climate change on planetary health. To this end, nurses must develop knowledge, expertise, evidence-based research, and partnerships that will enable them to contribute to raising environmental health for all populations [22]. Promoting environmental health helps limit exposure to hazardous agents and environmental conditions, thus promoting human well-being [23]. The nursing profession must imperatively combine its leadership role in developing and implementing effective health interventions with raising the population’s awareness of environmental threats to its health. This will require changes in personal and collective consciousness, as well as in behaviors and lifestyles so that they can all contribute to climate change adaptation and mitigation. In addition, at a more meta-level, nurses have a fundamental role to play in establishing health and environmental policies, which can contribute to increasing awareness of climate change and its consequences. The aim is to facilitate the implementation of various awareness-raising, prevention, and promotion initiatives, and to ensure their sustainability [24]. However, different studies [17,25,26] have shown that nurses’ knowledge about the health-related impacts of climate change, or their level of eco-literacy, was poor. The reasons revealed pertained to their sense of unpreparedness and perceived inability to address the impacts of climate change. Additionally, their moderate levels of awareness regarding its implications for both public health and planetary well-being were also brought to the fore. These parameters contribute to a lack of commitment to the environment from a profession that feels demotivated and overwhelmed because it does not know what to do about it and nursing research in this field is poorly developed [23]. Nurses are aware that they have the social and professional responsibility to address the health-related impacts of climate change, but very few of them think that their actions could have a significant effect [17,25,26]. There is currently no systematic review that identifies the most reliable, robust, and valid instruments for measuring nurses’ knowledge and awareness of climate change and climate-associated diseases. Additionally, of the three studies mentioned above, only Schenk et al. [26]’s study used an instrument with validated psychometric properties, the CHANT.

In recent years, eco-literacy has been considered the most important component of environmental education. The European Environment Information and Observation Network defines eco-literacy as the behaviors, attitudes, practices, and knowledge that society possesses concerning the maintenance and protection of its natural resources, the ecosystem, and all external conditions affecting human health. Thus, it includes knowledge and understanding of environmental concepts, problems, and issues, as well as cognitive and affective dispositions, skills, competencies, and appropriate behavioral strategies that help to implement this knowledge and enable informed decision-making [27]. An eco-literate citizen is not only more informed but also more capable of proactively and consciously solving or helping to solve environmental problems [28]. Knowledge associated with eco-literacy must include physical, ecological, social, cultural, and political systems, while a disposition to eco-literacy involves sensitivity, attitude, personal responsibility, and motivation. Finally, associated skills should include the ability to identify, analyze, investigate, evaluate, and resolve climate change consequences issues [27]. Eco-responsibility—a central component of eco-literacy—is the quality of a person or behavior that considers the principle of long-term respect for the physical, social, and economic environment [25]. This goes further than the accepted environmental metaparadigm that has led nurses to have egocentric and endemic blindness to climate change. They tend to focus on their immediate environment, with little consideration beyond the immediate context of the care they are providing; a more eco-centric perspective should be adopted. The knowledge derived from these concepts covers global health, climate health literacy, and even environmental health literacy.

Two main strategies are envisaged for dealing with climate change: (i) mitigation by actions that limit the extent and rate of climate change by constraining the emissions of greenhouse gases, and (ii) adaptation through initiatives and measures that can minimize the impacts of climate change and reduce its adverse effects on planetary health [25]. These strategies involve implementing behaviors aligned with planetary and population health [7]. New behaviors mean that raising nurses’ awareness, skills, and knowledge about these new climate-induced parameters becomes unavoidable. However, the literature has yet to identify the most reliable, robust, and valid instrument to do this. The present systematic review aims to identify the most reliable, robust, and valid instruments for measuring nurses’ knowledge and awareness of climate change and climate-associated diseases. In line with this, we have used the PCC (Population, Concept, and Context) (Table 1) method to develop our research questions and subsequently identify gaps in the literature. The review’s research questions are:

- What existing instruments are used to measure nurses’ knowledge and awareness of climate change and climate-associated diseases?
- How do the instruments identified vary in terms of their reliability, validity, and robustness for assessing nurses’ knowledge and awareness of climate change and climate-associated diseases?
- How do the different instruments address the multifaceted aspects of nurses’ knowledge and awareness of climate change and climate-associated diseases?
- What is the most reliable, robust, and valid instrument with which to measure nurses’ knowledge and awareness of climate change and climate-associated diseases?

To be agents of change, carry out health promotion and disease prevention initiatives, and offer adequate, effective, efficient, fair, safe, and patient-centered responses to patients’ needs, nurses will need to be ever more eco-literate. This is a way to encourage human adaptations and mitigations and to ensure that the nursing discipline is well-positioned to cope with climate change.

## 2. Materials and Methods

This systematic review protocol was registered in the PROSPERO international database of prospectively registered systematic reviews (protocol #: CRD42023407696). It will be conducted following the Preferred Reporting Items for Systematic Reviews and Meta-Analyses for Protocols (PRISMA-P) recommendations [29] and the Meta-analysis Of Observational Studies in Epidemiology (MOOSE) reporting proposals [30].

### 2.1. Eligibility Criteria

#### 2.1.1. Types of Studies

This review will include randomized controlled trials, cluster-randomized controlled trials, non-randomized studies, prospective cohort studies, case–control studies, controlled before-and-after studies, interrupted-time-series studies, and controlled trials with inappropriate randomization (quasi-experimental studies) [31,32]. We will consider publications in any language. DeepL Pro software and Google Translate will be used to translate the titles and abstracts for the studies selection. They were found to be viable tools for translating articles into English to facilitate data extraction and risk of bias assessment [33].

#### 2.1.2. Types of Participants

We will consider studies involving nursing students and registered nurses with bachelor’s, master’s, or doctoral degrees who are delivering primary healthcare services. The main mandate of primary healthcare is the provision of healthcare services, including the diagnosis and treatment of health conditions, and support for the management of long-term healthcare, including chronic conditions [10]. By focusing on primary healthcare services, we can quickly identify patients at risk of developing disorders that are the direct or indirect effects of climate change and offer them care tailored to their needs.

#### 2.1.3. Settings

We will include studies in all categories of healthcare and hospital settings and consider all institutions providing nursing training at the bachelor’s, master’s, and doctoral level.

#### 2.1.4. Types of Outcome Measures

The review’s primary outcome will be the identification of existing validated, reliable, and robust instruments measuring nurses’ knowledge and awareness of climate change and climate-associated diseases. The review’s secondary outcome will be a measure of nurses’ knowledge, awareness, motivation, attitudes, behaviors, beliefs, skills, and competencies regarding climate change and climate-associated diseases. As well as the variables that contribute to different levels of eco-literacy, as defined by the instrument.

#### 2.1.5. Statistical Analysis

In addition to a narrative synthesis, descriptive statistics will be used to describe the studies and participants involved. The statistical analysis plan for the quantitative variables will be used to describe the sample through central tendency (mean, median) and dispersion (IQR, the dispersion from 25% to 75%). If the criteria are met, a meta-analysis will be carried out. A fixed-effect or random-effect meta-analysis using the inverse-variance method and sensitivity analyses will be carried out depending on the type of data (dichotomous, continuous) and results. Methodological diversity, as well as statistical heterogeneity, will be measured [34]. Heterogeneity will be quantified using the I2 and chi-squared tests. Funnel plots will be drawn, and Egger tests will be computed to explore the possibilities of publication bias. To explore the possible determinants of heterogeneity, we will conduct subgroup analyses according to selected study characteristics (e.g., participants’ ages, the country where the study was conducted). Furthermore, sensitivity analyses will be calculated by: (1) excluding relatively small studies (with fewer than 20 participants per randomization group, and (2) restricting analyses to the best-quality studies. These data will also be analyzed using SPSS software, version 29.0, and Review Manager, version 5.5.

### 2.2. Inclusion and Exclusion Criteria

Inclusion and exclsuion criteria applied to the studies are presented in Table 1.

### 2.3. Search Strategy

In collaboration with a medical librarian (PM) and using predefined search terms, we will conduct a systematic literature search for published articles in the following electronic databases, from inception until 31 October 2023: Medline Ovid SP (from 1946), PubMed (NOT Medline[sb]; from 1996), Embase.com (from 1947), CINAHL Ebesco (from 1937), the Cochrane Library Wiley (from 1992), Web of Science Core Collection (from 1900), the Trip Database (from 1997), JBI OVID SP (from 1998), and the GreenFILE EBSCO. We will also conduct a hand-search of all the relevant articles’ bibliographies and search for unpublished studies using Google Scholar, ProQuest Dissertations and Theses Global, and DART-EUrope.eu. A new search will be carried out on 31 October to ensure that articles published between May and October are taken into consideration.

The search syntax used to investigate these databases will serve as the basis for all our search strategies, using descriptors (EMTREE and Medical Subject Headings [MeSH]) and text terms with the Boolean operators “AND” and “OR”. The syntax consists of the following four search themes, intersected by the Boolean terms “AND” and “OR”: nurses, climate change, literacy, and scales/tools. (Table 2).

### 2.4. Data Collection and Analysis

#### 2.4.1. Study Selection

Two reviewers (HV and OPDS) will independently screen the titles and abstracts identified in the searches to assess which studies meet the inclusion criteria. Disagreements will be resolved through discussion or, if needed, a consensus will be reached after discussion with a co-author (SJ).

Two reviewers (HV and OPDS) will independently assess the full-text articles identified to ensure they meet the inclusion criteria. Any disagreement about the quality evaluations will be resolved through discussion or, if needed, a consensus will be reached after discussion with a co-author (SJ). The results of this screening exercise will be reported in a PRISMA flow diagram [29]. The relevant articles retained for the review will be compiled, and duplicate records will be identified using EndNote reference manager software.

#### 2.4.2. Data Extraction

Data extraction will be conducted independently by two authors (HV and OPDS) using a specially designed, standardized data extraction form. Discrepancies will be resolved through discussion and consultation with a co-author (SJ). The following information will be extracted from each study included: (i) study authors, year of publication, and country where the study was conducted; (ii) study characteristics (including setting and design, duration of follow-up, and sample size); (iii) participants’ characteristics (e.g., employment [% vs. hours/week], employer, sex, age); (iv) psychometric properties (e.g., Cronbach’s alpha, Cohen’s Kappa, intra-class correlation coefficient, reliability, validity, fidelity, coefficient reliability, standard error measurement, limits of agreement, confirmatory factor analysis, number of items, number of domains, type of response scale, scale, subscale); and (v) types of outcome measures.

#### 2.4.3. Assessment of the Risks in the Studies Included

A multi-method approach will be employed, with the JBI critical appraisal tool used to evaluate the methodological quality and the Consensus-based Standards for the selection of health Measurement Instruments (COSMIN) recommendations used to assess the relevance of the instruments in studies with a psychometric or clinometric orientation. Two reviewers (HV and OPDS) will independently assess the risks of bias in all the randomized and non-randomized studies of interventions included. Conflicts will be addressed by engaging in discussions and seeking a resolution through input from a co-author (JS). Different tools will be used to assess the studies’ methodological quality. The Newcastle–Ottawa Scale for assessing the quality of non-randomized studies in meta-analyses [35] will be used to assess cohort studies. The validated Risk Of Bias In Non-randomized Studies of Interventions (ROBINS-I) tool [36] will be used to evaluate quasi-experimental designs. Finally, the Revised Cochrane Risk of Bias tool will be used to assess randomized trials (RoB 2.0) [37]. Two researchers (OPDS and HV) will independently rate the studies’ quality. Any disagreements about quality assessments will be resolved by discussion.

The measurement of health outcomes is essential in both scientific research and clinical practice. Instruments must be reliable and valid. Two researchers will evaluate the measurement instrument’s various psychometric properties, such as reliability (internal consistency, test–retest reliability), validity (content, construct, criterion), responsiveness, and interpretability. The COSMIN recommendations evaluate the methodological quality of studies on different measurements’ properties [38]. The COSMIN taxonomy considers cross-sectional measurements (reliability and validity) and longitudinal measurements (the reliability of change scores and responsiveness) [39]. Two researchers (OPDS and HV) will use the COSMIN Risk of Bias tool to evaluate the various psychometric properties of each measurement instrument, such as reliability (internal consistency, test–retest reliability), validity (content, construct, criterion), responsiveness, and interpretability. They will assess the development of patient-related outcome measures (PROMs), content validity, structural validity, internal consistency, cross-cultural validity or measurement invariance, reliability, measurement error, criterion validity, hypotheses testing for construct validity, and responsiveness [40]. Finally, the COSMIN Risk of Bias tool will be used to assess the quality of studies’ reliability and measurement instruments’ measurement error of outcomes [40].

## 3. Results

The search strategy selected will begin with two researchers independently analyzing the titles and abstracts (Table 3). An artificial intelligence-based algorithm named Deduklick will identify and remove duplicate references [41]. In the second phase, full-text papers will be retrieved from the references and analyzed based on the inclusion and exclusion criteria identified. Finally, all the full-text articles retained that meet our criteria will be analyzed and reported in a structured, systematic literature review. Results are expected in October 2023.

## 4. Discussion

To the best of our knowledge, no systematic reviews have attempted to identify tools that measure nurses’ knowledge and awareness of climate change and climate-associated diseases. This systematic review research protocol will inform us about existing validated, reliable, and robust instruments measuring nurses’ knowledge and awareness of these topics [27]. The nursing discipline has always recognized the environment’s importance, even though its definition and impact on health have varied according to different paradigms, theories, models, and time [23]. Once nurses’ levels of eco-literacy have been assessed, we will be able to propose interventions to boost their knowledge, awareness, motivation, attitudes, behaviors, beliefs, skills, and competencies about climate change and climate-associated diseases. These improvements will allow them to become agents of change and to expand or improve the roles they play as educators, influencers, and informed advocates supporting patients, families, and communities. In addition, at a more meta-level, nurses have a fundamental role to play in the establishment of health and environmental policies, which can contribute to increasing awareness of climate change and its consequences. Indeed, they will be able to apply their knowledge and awareness of climate change and climate-associated diseases, with a patient-centered approach, to decision-making at a political, organizational, and institutional level. They will ensure that resources and means are used wisely to enable mitigation and adaptation. Nursing is therefore facing a major challenge and must take the most appropriate response to this global problem Furthermore, the results of our eco-literacy assessment of registered nurses providing direct care to patients could be used to inform future research projects and guide policymakers, stakeholders, and healthcare professionals about changes to include in undergraduate and graduate nursing curricula. It would ensure that current and future generations of nurses will be able to meet the population’s needs and provide adequate, effective, efficient, fair, safe, and patient-centered responses to patients’ care needs with regard to the consequences of climate change. This, in turn, will improve the nursing profession’s commitment and motivation to develop, implement, and sustain innovations in the field of climate change mitigation and adaptation [42,43].

## Figures and Tables

**Table 1:** Study inclusion and exclusion criteria.

PCC	Inclusion Criteria	Exclusion Criteria
Population	- Studies on nursing students and registered nurses with bachelor’s, master’s, or doctoral degrees who are delivering primary healthcare services.	- Studies that focus on nurses who no longer provide direct care (head nurse, manager, etc.). - Studies that do not include measures of nurses’ knowledge and awareness of climate change and climate-associated diseases.
Concept	- Studies whose primary objective is to assess or report on the measurement properties of instruments used to gauge nurses’ knowledge and awareness of climate change and climate-associated diseases. - Studies that examine instruments to measure nurses’ knowledge and awareness of climate change and climate-associated diseases. - Studies that directly address climate change and its associated diseases as the subject matter of measurement. - Studies that evaluate the psychometric properties of the measurement instruments, including, but not limited to, reliability (internal consistency, test–retest reliability), validity (content, construct, criterion), responsiveness, and interpretability.	- Studies that do not contain data on nursing students and registered nurses with bachelor’s, master’s, or doctoral degrees who are delivering primary healthcare services. - Studies that do not specifically address measurement instruments designed to assess nurses’ knowledge and awareness of climate change and climate-associated diseases. - Reviews, commentaries, opinion pieces, and editorials that do not provide original empirical data on the measurement instruments. - Studies that do not provide sufficient methodological details to assess the study’s quality or the instrument’s measurement properties.
Context	All contexts will be considered	

**Table 2:** Search Syntax Strategy.

	Climate Change	Literacy	Nursing	Tools
Free words	((climat* NEAR/1 (chang* OR variabilit* OR sensitivity OR warming)) OR “greenhouse effect*” OR “greenhouse gas” OR “global warming” OR “extreme weather” OR “severe weather” OR heatwave* OR coldwave OR ((hot OR cold OR heat) NEAR/1 (wave* OR snap* OR spell* OR extreme)) OR (Environmental NEXT (pollution* OR impact* OR health)) “air pollution*” OR “air quality” OR	((information OR health NEXT/1 literac*) OR Knowledge OR (nurse* NEXT/1 role*) OR Attitude* OR Behavior* OR Motivation* OR Competenc* OR Skill* OR Expertise OR Awareness OR Comprehension OR Belief* OR empowerment	nurse* OR nursing	psychometr* OR measure* OR instrument* OR tool$ OR toolkit* OR questionnaire* OR survey* OR interview* OR “G theory” OR “generali?ability theory”
EMTREE	‘climate change’/exp OR ‘greenhouse effect’/exp OR ‘climate’/de OR ‘extreme weather’/exp OR ‘heat wave’/exp OR ‘cold wave (weather)’/exp OR ‘severe weather’/de OR ‘pollution’/de OR ‘air pollution’/de OR ‘environment’/de OR ‘environmental health’/de	‘health literacy’/exp OR ‘information literacy’/exp OR ‘knowledge’/exp OR ‘attitude’/de OR ‘health personnel attitude’/de OR ‘nurse attitude’/de OR ‘behavior’/de OR ‘empowerment’/exp OR ‘motivation’/exp OR ‘competence’/exp OR ‘awareness’/exp OR ‘comprehension’/exp	‘nurse’/exp OR ‘nursing’/exp OR ‘nursing student’/exp	‘reproducibility’/exp OR ‘psychometry’/exp OR ‘interview’/exp OR ‘questionnaire’/exp
MeSH	“Climate Change”[Mesh] OR “Greenhouse Effect”[Mesh] OR “Climate”[Mesh:NoExp] OR “Extreme Weather”[Mesh] OR “Extreme Heat”[Mesh] OR “Environmental Pollution”[Mesh:NoExp] OR “Air Pollution”[Mesh:NoExp] OR “Environment”[Mesh:NoExp] OR “Environmental Health”[Mesh]	“Information Literacy”[Mesh] OR “Knowledge”[Mesh] OR “Attitude”[Mesh:NoExp] OR “Attitude of Health Personnel”[Mesh:NoExp] OR “Nurse’s Role”[Mesh] OR “Behavior”[Mesh:NoExp] OR “Motivation”[Mesh] OR “Professional Competence”[Mesh] OR “Awareness”[Mesh] OR “Empowerment”[Mesh] OR “Comprehension”[Mesh]	“Nurses”[Mesh] OR “Nursing”[Mesh]	“Reproducibility of Results”[Mesh] OR “Psychometrics”[Mesh] OR “Surveys and Questionnaires”[Mesh] OR “Interviews as Topic”[Mesh]
CINAHL	(MH “Climate+” OR MH “Extreme Weather” OR MH “Heat” OR MH “Cold” OR MH “Environmental Pollution” OR MH “Air Pollution” OR MH “Environment” OR MH “Environmental Health”	MH “Information Literacy+” OR MH “Knowledge+” OR MH “Attitude” OR MH “Attitude of Health Personnel” OR MH “Nurse Attitudes” OR MH “Behavior” OR MH “Empowerment” OR MH “Motivation” OR MH “Professional Competence+” OR MH “Cognition”	MH “Information Literacy+” OR MH “Knowledge+” OR MH “Attitude” OR MH “Attitude of Health Personnel” OR MH “Nurse Attitudes” OR MH “Behavior” OR MH “Empowerment” OR MH “Motivation” OR MH “Professional Competence+” OR MH “Cognition”	MH “Reproducibility of Results” OR MH “Psychometrics” OR MH “Interviews+” OR MH “Interview Guides+” OR MH “Questionnaires+” OR MH “Surveys+”
All term with “*”means that means that all the terms in the same truncation are added together				

**Table 3:** Number of references retrieved using our search strategy.

Sources	Search Date	Total Number of References Found	After Removing Duplicates
Databases	28 May 2023		
Pubmed		25	3
Medline OVID SP		411	411
Embase.com		1402	1085
CINAHL Ultimate EBSCO		392	242
Cochrane Library Wiley		18	10
Web of Science-Core Collection		322	160
JBI OVID SP		39	38
GreenFILE EBSCO		36	11
Total		2645	1960
Other sources			
DART-Europe.eu		51	51
ProQuest Dissertations and Theses		6	6
TRIP database.com		1232	1232
Google Scholar		200	200
Total		1489	1489
Results		4134	3449
